# The DNA helicase HELQ promotes replication fork reversal in coordination with BRCA2- and FANCD2-mediated repair pathways

**DOI:** 10.1093/nar/gkag332

**Published:** 2026-04-29

**Authors:** Yerkin Dunbayev, Yen-Ju Chen, Lorenzo Sassi, Eun A Lee, Jae Sun Ra, Moonjung Choi, Anirban Mukherjee, Karen M Vasquez, Vincenzo Costanzo, Peter Chi, Kei-ichi Takata

**Affiliations:** Center for Genomic Integrity, Institute for Basic Science, Ulsan 44919, Republic of Korea; Department of Biological Sciences, Ulsan National Institute of Science and Technology, Ulsan 44919, Republic of Korea; Institute of Biochemical Sciences, National Taiwan University, Taipei 10617, Taiwan; Department of Oncology and Hematology-Oncology, University of Milan, Via Adamello 16, Milan 20139, Italy; IFOM-ETS, The AIRC Institute of Molecular Oncology, Milan 20139, Italy; Center for Genomic Integrity, Institute for Basic Science, Ulsan 44919, Republic of Korea; Center for Genomic Integrity, Institute for Basic Science, Ulsan 44919, Republic of Korea; Center for Genomic Integrity, Institute for Basic Science, Ulsan 44919, Republic of Korea; Division of Pharmacology and Toxicology, College of Pharmacy, The University of Texas at Austin, Dell Pediatric Research Institute, 1400 Barbara Jordan Boulevard, Austin, TX 78723, United States; Division of Pharmacology and Toxicology, College of Pharmacy, The University of Texas at Austin, Dell Pediatric Research Institute, 1400 Barbara Jordan Boulevard, Austin, TX 78723, United States; Department of Oncology and Hematology-Oncology, University of Milan, Via Adamello 16, Milan 20139, Italy; IFOM-ETS, The AIRC Institute of Molecular Oncology, Milan 20139, Italy; Institute of Biochemical Sciences, National Taiwan University, Taipei 10617, Taiwan; Institute of Biological Chemistry, Academia Sinica, Taipei 11529, Taiwan; Center for Genomic Integrity, Institute for Basic Science, Ulsan 44919, Republic of Korea; Department of Biomedical Engineering, College of Information-Bio Convergence Engineering, Ulsan National Institute of Science and Technology, Ulsan 44919, Republic of Korea

## Abstract

HELQ is a 3′–5′ DNA helicase whose loss sensitizes cells to DNA-damaging agents, particularly DNA crosslinkers. HELQ interacts with the RAD51 paralog complex RAD51B–RAD51C–RAD51D–XRCC2 (BCDX2), a key mediator of replication fork reversal. Using DNA fiber assays, we show that HELQ and BCDX2 act epistatically to slow replication fork progression under replication stress. Because fork reversal transiently regresses nascent strands into a four-way junction and reduces net DNA synthesis, this fork slowing provides a functional readout of fork reversal. Directly supporting this model, electron microscopy reveals that reversed fork structures are reduced in HELQ-knockout cells. Consistent with a role in fork reversal, HELQ deletion suppresses nascent strand degradation when BRCA2- or FANCD2-dependent fork protection is lost. Mechanistically, biochemical reconstitution shows that HELQ is stimulated by RPA on fork substrates containing a leading strand gap, and these findings are consistent with the cell-based DNA fiber assays. Together, these results identify HELQ as a specialized regulator of replication fork remodeling that promotes fork reversal through the BCDX2 pathway.

## Graphical Abstract

**Graphical Abstract:**
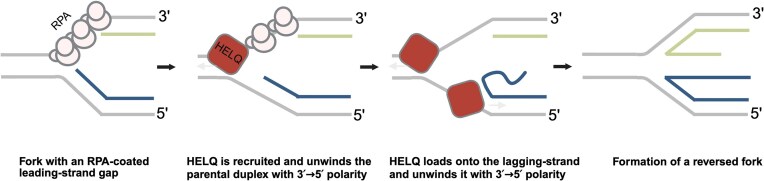

## Introduction

DNA synthesis is essential for genome duplication, but the replicative DNA polymerases frequently encounter obstacles that can impede their progression, leading to replication fork stalling. These obstacles include DNA damage introduced by endogenous sources such as water-catalyzed hydrolysis and reactive oxygen species (ROS) and by environmental sources including ionizing radiation (IR), ultraviolet (UV), and DNA interstrand crosslink (ICL)-inducing reagents such as mitomycin C and cisplatin. The toxic and mutagenic consequences of DNA lesions are minimized by distinct DNA repair pathways [1]. When DNA damage persists, cells engage DNA damage tolerance mechanisms, including fork remodeling, translesion synthesis (TLS), and repriming by PRIMPOL [2, 3].

Uncoupling of the replicative helicase and polymerase at DNA lesions generates extended regions of RPA-bound ssDNA behind the replication fork [4]. These structures can promote replication fork reversal, a remodeling reaction primarily driven by DNA translocases SMARCAL1, ZRANB3, HLTF, and FBH1, along with RAD51, RAD51 paralog complex BCDX2 (RAD51B–RAD51C–RAD51D–XRCC2), and RECQ5 [2, 5, 6]. Reversed forks are protected by RAD51, BRCA1/2, FANCD2, BCDX2, and 53BP1 and loss of these factors leads to degradation of nascent strands by nucleases such as MRE11, EXO1, and DNA2 [7–9]. At least two fork protection mechanisms have been described: BRCA2 and FANCD2 protect reversed forks generated by SMARCAL1, ZRANB3, and HLTF, while 53BP1 protects forks reversed by FBH1 [10]. Fork remodeling is thought to provide sufficient time for cells to repair DNA lesions. Subsequently, reversed forks are restarted by RECQ1 and another RAD51 paralog complex X3CDX2 (XRCC3–RAD51C–RAD51D–XRCC2) [5, 11]. RAD51 paralogs assemble into higher-order subcomplexes, including BCDX2 and X3CDX2, which carry out distinct functions in homologous recombination (HR) and replication fork remodeling. All five paralogs contribute to efficient RAD51 foci formation after DNA damage in human cells [12], and by selectively assembling RAD51 on ssDNA, they limit inappropriate filament formation on dsDNA, a known source of genomic instability [13]. Within this network, the BCDX2 complex operates as a recombination mediator that promotes RAD51 filament assembly on RPA-coated ssDNA via an ATP hydrolysis–dependent cycle of high- and low-affinity ssDNA binding, thereby facilitating filament nucleation and extension [14, 15]. In contrast, the X3CDX2 complex targets RAD51 to RPA-free ssDNA, lacks ATPase coupling, and forms a stable cap at the 5′ termini of RAD51 filaments, thereby enhancing their strand-exchange activity. X3CDX2, but not BCDX2, stimulates RAD51-mediated D-loop formation [11]. These distinct activities of BCDX2 and X3CDX2 are thought to be critical for promoting replication fork reversal and fork restart, respectively, during the cellular response to replication stress [5]. If stalled forks are not processed in a timely manner, they can be converted into DNA double-strand breaks (DSBs) by the nuclease activity of MUS81, thereby promoting mutagenic restart via break-induced DNA replication (BIR) [16, 17].

TLS and repriming constitute additional DNA damage tolerance mechanisms. Specialized TLS polymerases such as Pol η, Pol κ, and Pol ι insert nucleotides opposite damaged bases, allowing replication forks to bypass lesions and continue DNA synthesis; however, this process is frequently mutagenic and has been linked to the accumulation of point mutations observed in cancer genomes [18]. By contrast, PRIMPOL promotes DNA repriming downstream of replication-blocking lesions, thereby enabling fork restart at the expense of generating post-replicative gaps [19], and also participates in a specialized ICL repair pathway known as ICL traverse [20]. When these tolerance pathways fail, replication forks collapse, giving rise to DSBs and consequent chromosome rearrangements characteristic of many cancer cells [18]. Because replication fork dynamics are critical determinants of cellular responses to chemotherapeutic agents, elucidating the mechanisms that govern DNA damage tolerance has the potential to inform improved strategies for cancer prevention and treatment.

HELQ is a DNA helicase that translocates in the 3′–5′ direction and can unwind duplex DNA [21]. Based on biochemical studies using model replication fork substrates, HELQ has been proposed to open the parental strands at blocked DNA replication forks and to displace nascent lagging strand intermediates, thereby facilitating the loading of downstream factors required for DNA damage processing or replication restart [22, 23]. Nevertheless, the precise role of HELQ at stalled replication forks in cells remains poorly understood. HELQ contributes to the protection of human cells from DNA damage, particularly ICLs [24–26], and interacts with the RAD51 paralog complex BCDX2 but not with the XRCC3-containing complex X3CDX2 [25, 26]. However, the biological significance of this selective interaction has not yet been clarified. Given that BCDX2 participates in fork reversal [5] and that fork remodeling is required for DNA damage tolerance and efficient ICL repair [17, 27], we hypothesized that HELQ cooperates with BCDX2 to promote fork remodeling. Here, we identify a previously unrecognized role for HELQ in replication-mediated fork remodeling.

## Materials and methods

### Cell culture and cell lines

Human U2OS and human lung cancer A549 cells were grown in Dulbecco’s modified Eagle’s medium (DMEM) supplemented with 10% fetal bovine serum (FBS; Gibco/Thermo Fisher Scientific) and 1% penicillin–streptomycin antibiotics (100 U/ml penicillin and 100 μg/ml streptomycin) under standard cell culture conditions (humidified atmosphere, 5% CO_2_) at 37°C. *HELQ*-deficient U2OS cells have been generated and genetically characterized as recently reported [25]. *XRCC3*-deficient U2OS; *HELQ*-deficient and HELQ K365M mutant A549 cells were generated as follows. Custom oligonucleotides targeting the XRCC3 or HELQ gene were designed for Thermo Fisher GenArt CRISPR system according to manufacturer’s instructions based on designs from previous publications [12] (XRCC3 top strand: 5’-GGTCTGGCACTTGCTGAGAAGTTTT; XRCC3 bottom strand: 5’-TTCTCAGCAAGTGCCAGACCCGGTG; HELQ knockout top strand: 5’-CTCAACCGGCAGTACGCCCGGTTTT; HELQ knockout bottom strand: 5’-CGGGCGTACTGCCGGTTGAGCGGTG; HELQ K365M knock-in top strand: 5’-AAGTGGTGGAAAAACCCTCGGTTTT; HELQ K365M knock-in bottom strand: 5’-CGAGGGTTTTTCCACCACTTCGGTG). For generation of the HELQ K365M knock-in mutant, an ssODN donor oligonucleotide (5′-CTTTTCTTGGACAATTGCCACATATGGAAGAATCATTAAAACATCTTTCCGACAGCAAAGCAGTTCTTGCAGCATTAAAATCTCAGCCACGAGGGTCATTCCACCACTTGTTGGCAAGGAATATATTAAATTTTTTCTTTCTTGCACAGAATTCAATGTTAAACAAGTATGTTGCCATTCTGTGGAATT) was co-transfected to replace the lysine codon at position 365 (AAA) with a methionine codon (ATG; underlined). Transfection of the CRISPR vector was done using Thermo Fisher Neon transfection system. After the transfection, single-cell clones were sorted into 96-well plates by flow cytometry. Genomic DNA isolated from individual clones was amplified by PCR. For confirmation of XRCC3 knockout, forward (5′-TGCGTTGTGACAGTCTGACA) and reverse (5′-TTCTGACCCGATGCTGTGAC) primers were used, and then each PCR product was used for the Cel-I Nuclease Mismatch assay (Surveyor). For confirmation of the K365M mutation, forward (5′-GAGTTTCAAAGTTTGGAAGGGGAAA) and reverse (5′-ATACACACACACACACCTTTTCTTG) primers were used. The targeted genomic DNA sequences were verified by TA-cloning and sequencing.

### siRNA transfection

The siRNAs used in this study were siControl (Horizon Discovery, ON-TARGETplus Non-targeting pool, D-001810-10-05), siPRIMPOL (Bioneer, custom order; sense sequence: 5′-AGAAAAGGCUACAGAGGAAtt, where the terminal tt residues are DNA), siSMARCAL1 (Horizon Discovery, ON-TARGETplus, L-013058-00-0005), siZRANB3 (Thermo Fisher, Stealth RNAi, custom order; sense sequence: 5′-UGAUUCUCAGAAAGACACCUCCAAA), siBRCA2 (Horizon Discovery, ON-TARGETplus, L-003462-00-0005), siFANCD2 (Bioneer, custom order; sense sequence: 5′-AAAAUGAGUCGAGGUAUGUUG), siXRCC2 (Thermo Fisher, Silencer Select, custom order; sense sequence: 5′-UUCUUUUUGCAACGACACAAACUAU), and siXRCC3 (Thermo Fisher, Silencer Select, custom order; sense sequence: 5′-AUCCCAGAAUUAUUGCUGCAAUUAA). For custom-ordered siRNAs, the sense strand sequences are listed, whereas catalog numbers are provided for commercially available siRNAs. The siRNAs were introduced into wild-type or *HELQ* knockout U2OS cells. Twenty-four hours prior to transfection, cells were plated in a 100 mm plate at 1.0 × 10^6^ cells/well. For each well, 15 pmol of siRNAs was diluted in 750 μl of Opti-MEM (Invitrogen). In a separate tube, 15 μl of Lipofectamine RNAiMAX reagent (Invitrogen) was diluted in 750 μl of Opti-MEM and incubated at room temperature for 10 min. The Lipofectamine RNAiMAX dilution was added into the diluted siRNA duplex and incubated at room temperature for 20 min. Before the transfection, medium was replaced with fresh 7.5 ml DMEM supplemented with 10% fetal bovine serum for each well. The Lipofectamine RNAiMAX-siRNA complex was added dropwise to the cells and incubated at 37°C. After 24 h, the cells were washed, trypsinized, and plated with fresh DMEM medium supplemented with 10% fetal bovine serum and 1% penicillin–streptomycin (Invitrogen). To determine the protein levels of proteins, whole-cell crude extracts were prepared 48 h after the RNA transfection and analyzed by immunoblotting.

### Drugs and reagents

Hydroxyurea (HU; H8627, Sigma–Aldrich) was prepared in double-distilled water to obtain a 1 M stock and stored at −20°C; Camptothecin (CPT; C9911, Sigma–Aldrich) was dissolved in DMSO to yield a 5 mM stock, and aliquots were stored at −20°C; BrdU was dissolved in double-distilled water to yield 1 M stock, and aliquots were stored at −20°C; Thymidine (T9250, Sigma−Aldrich) was dissolved in double-distilled water to yield 100 mM stock and used fresh; Mitomycin C (MMC, M4287, Sigma−Aldrich) was dissolved in double-distilled water to yield 1.5 M stock and used fresh. Mirin (475954-10MGCN, Sigma-Aldrich) was dissolved in DMSO to yield 5 mg/ml stock and aliquots were stored at -20 °C. Pol α inhibitor CD437 (C5865, Sigma−Aldrich) was dissolved in DMSO to yield 5 mM stock and aliquots were stored at −20°C.

### Immunoblotting

Cells were washed in cold PBS and lysed in RIPA buffer (50 mM Tris–HCl [pH 7.5], 150 mM NaCl, 1% NP-40) supplemented with 1× protease inhibitor cocktail (cOmplete, Roche) and phosphatase inhibitors (20 mM NaF, 1 mM Na_3_VO_4_ and 5 mM Na_4_P_2_O_7_). Cell extracts were diluted with NuPAGE LDS sample buffer (Thermo Fisher Scientific) containing DTT and heated at 55°C for 5 min. Proteins, together with the PageRuler Plus Prestained Protein Ladder (Thermo Fisher Scientific, 26620), were resolved on NuPAGE 3–8% Tris-Acetate gels for BRCA2 and FANCD2, and on NuPAGE 4–12% Bis-Tris gels for all other proteins (Thermo Fisher Scientific), using NuPAGE Tris-Acetate SDS running buffer and NuPAGE MOPS SDS running buffer, respectively, and transferred onto nitrocellulose membranes for BRCA2 and FANCD2, and PVDF membranes for all other proteins. Membranes were blocked with 10% skim milk powder dissolved in TBS containing 0.1% Tween-20 for 2 h at room temperature (RT), incubated with the indicated primary antibodies overnight at 4°C and secondary antibodies for 1 or 2 h at RT. Proteins were visualized using the Amersham ECL Prime/Pierce ECL Plus reagent and the ChemiDoc Touch imaging system. The following primary antibodies were used: XRCC2 (sc-365854, Santa Cruz Biotechnology, 1:200), XRCC3 (sc-271714, Santa Cruz Biotechnology, 1:200), BRCA2 (ab9143, Abcam, 1:1000), ZRANB3 (23111-1-AP, Proteintech, 1:500), FANCD2 (sc20022, Santa Cruz, 1:1000), α-Tubulin (T5168, Sigma–Aldrich, 1:5000), Vinculin (sc-73614, Santa Cruz, 1:5000), pSer345 CHK1 (133D3, Cell Signaling, 1:1000), HELQ (D4K2O, Cell Signaling, 1:1000), CHK1 (sc-8408, Santa Cruz, 1:2000), SMARCAL1 (SAB2107964, Sigma–Aldrich, 1:1000), pSer345 CHK1 (133D3, Cell Signaling, 1:1000), pSer33 RPA2 (A300-246A, Bethyl, 1:1000), ATM (ab32420, Abcam, 1:1000), pSer1981 ATM (ab81292, Abcam, 1:5000), CHK2 (2662, Cell Signaling, 1:500), and pThr68 CHK2 (2197, Cell Signaling, 1:1000).

### DNA fiber assays

DNA combing was performed as previously described [28]. In brief, cells were transfected with corresponding siRNA for 24 h, seeded into 60 mm dishes, and grown for 24 h. Next, they were labeled with the thymidine analogs 5-chloro-2′-deoxyuridine (CldU, 100 μM), washed three times with PBS, followed by 5-iodo-2′-deoxyuridine (IdU, 250 μM), and treated with HU, MMC, cisplatin, and CPT as indicated. In the case of psoralen-UVA treatment, cells were washed once with PBS before UVA irradiation (1 J/m^2^) in a UVA chamber. 300 000 cells were used to form a DNA plug, and cell lysis was performed overnight. CldU was detected using an anti-BrdU antibody (Abcam ab6326, 1:100 dilution); IdU was detected using different anti-BrdU antibody (BD Biosciences 347580, 1:100 dilution). Slides were mounted, and image acquisition was done using an inverted fluorescence microscope (Axio Observer 7, Carl Zeiss) with 63× objective, oil. Track lengths were measured using Zen Blue distance tool, with at least 100 tracks per set measured. Kruskal–Wallis test analysis was performed using Prism software. Experiments were repeated at least three times.

### Fork reversal assay

DNA substrates were prepared and quantified using absorbance at 260 nm. For the fork reversal assay, the indicated amount of HELQ protein was added to the reaction buffer (35 mM Tris–HCl, pH 7.5, 50 mM KCl, 2 mM Mg^2+^, 2 mM ATP, and 0.1 mg/ml BSA) with DNA substrates (8 nM) in 10 μl final volume at 37°C for 30 min. The reaction was terminated by adding 2.5 μl of termination buffer [with final 48 mM ethylenediaminetetraacetic acid (EDTA), 0.02% SDS, and 0.64 mg/ml proteinase K] and incubated at 37°C for 15 min. Subsequently, 3 μl of loading buffer (20 mM Tris–HCl, pH 7.5, and 50% glycerol) was added, and the samples were analyzed using 6% TBE–PAGE. Gels were imaged using an Amersham™ Typhoon™ Biomolecular Imager (GE Healthcare) with a Cy3 570BP20 560–580 nm filter. Signal intensity of DNA species was quantified using Amersham™ ImageQuant™ software. To test the stimulation of BCDX2 on HELQ, HELQ was pre-incubated with BCDX2 in reaction buffer on ice, then DNA substrates (8 nM) were added to initiate the reaction at 37°C for 20 min. To test the stimulation of RPA on HELQ, HELQ was pre-incubated with RPA in reaction buffer on ice, and then leading strand gap DNA substrates (16 nM) were added to initiate the reaction at 37°C for 20 min. The reaction was terminated and analyzed as the same procedure described earlier.

### Fork protection assay

The procedure for the MRE11 protection assay aligns with methods previously reported [8]. Briefly, a 29 nM fluorescently labeled 5′ overhang DNA substrate was pre-incubated with HELQ and RAD51 in 10 μl of reaction buffer (35 mM Tris–HCl, pH 7.5, 1 mM DTT, 50 mM KCl, 2.5 mM Mg^2+^, 1 mM ATP, 1 mM MnCl_2_, and 0.1 mg/ml BSA) at 37°C for 10 min. Subsequently, purified human MRE11 was added and allowed to digest the DNA for 40 min. The reaction was terminated by adding 2.5 μl of termination buffer and incubating at 37°C for 15 min. An equal volume of 2× denaturing dye (95% formamide, 0.1% Orange G, 10 mM Tris–HCl, pH 7.5, 1 mM EDTA, and 12% Ficoll PM400) was then added, and the mixture was incubated at 95°C for 10 min to denature the DNA substrates. The samples were analyzed on a 27% denaturing TBE–Urea–PAGE (7 M Urea) using 1× TBE buffer at 300 V for 40 min at 55°C. Gels were imaged using an Amersham™ Typhoon™ Biomolecular Imager with a Cy3 570BP20 560–580 nm filter. Amersham™ ImageQuant™ software was utilized to quantify the signal intensity of the DNA species.

### Replication intermediate transmission electron microscopy

Overall, the procedure followed previously established protocol [29] with adjustments as described. After treating with 1 μM camptothecin for 30 min, 10 × 10^6^ G1/S synchronized U2OS cells were washed with PBS and then harvested by trypsinization and resuspended in 10 ml of cold PBS. Afterwards, DNA was crosslinked by treating with 10 μg/ml final concentration 4,5′,8-trimethylpsoralen (TMP) and irradiating with four 10-min 365 nm UVA monochromatic light pulses (UVP Crosslinker CL1000L). This step was repeated one more time before proceeding further. Next, crosslinked samples were washed three times with cold PBS and lysed using a cell lysis buffer [1.28 M sucrose, 40 mM Tris–HCl, pH 7.5, 20 mM MgCl_2_, and 4% (v/v) Triton X-100]. The nuclei were then digested in a digestion buffer [800 mM guanidine-HCl, 30 mM Tris–HCl, pH 8.0, 30 mM EDTA, pH 8.0, 5% (v/v) Tween 20, and 0.5% (v/v) Triton X-100] supplemented with 125 μl of 10 mg/ml RNase A at 37°C for 2 h, and then 1 mg/ml proteinase K was added and further incubated at 50°C for 2 h. Genomic DNA was extracted with a 25:24:1 phenol:chloroform:isoamyl alcohol mixture by phase separation (centrifugation at 8000 rcf for 20′ at 4°C), further purified by 24:1 chloroform:isoamyl alcohol clean-up and precipitated by addition of equal amount of isopropanol to the aqueous phase, followed by another centrifugation step (8000 rcf for 10′ at 4°C). The obtained DNA pellet was washed once with 1 ml of 70% ethanol, air-dried at RT, and resuspended by overnight incubation in 200 μl TE (Tris–EDTA) buffer at RT. 10 μg of the extracted genomic DNA was digested for 5 h at 37°C with 50 U of restriction enzyme PvuII-HF (#R3151S, New England Biolabs). Digestion mixture was then cleaned up by loading onto Qiagen Genomic-Tip 20/G column (Qiagen, 10223) and eluting using 1200 μl of 10 mM Tris–HCl pH 8.0, 1 M NaCl, and 1.8% (w/v) caffeine. The eluate was concentrated by spinning in Amicon Ultra-0.5 Centrifugal Filter Units with Ultracel-100 (Millipore, UFC510096). Subsequent manipulations have been performed as described before [29, 30]. Images were acquired at the IFOM EM facility using a Thermo Fisher TALOS F200C transmission electron microscope equipped with a Ceta-S camera.

### Expression and purification of human HELQ

pcDNA3.4-(His)_6_-flag-HELQ was transfected into Expi293F cells according to the instruction manual from the ExpiFectamine 293 kit (Thermo Fisher Scientific). Cells were harvested by centrifugation after 48 h transfection. All protein purification steps were carried out at 4°C. Cell pellets were resuspended in buffer A (25 mM Tris–HCl, pH 7.5, 10% glycerol, 500 mM KCl, 0.01% Igepal-CA-630, 0.5 mM EDTA, 10 mM ATP, and 10 mM MgCl_2_) containing 1 mM β-mercaptoethanol and protease inhibitors (2 mM PMSF, 2 mM benzamidine and 1 μg/ml each of aprotinin, chymostatin, leupeptin, and pepstatin A). The resuspended cells were lysed by sonication and then centrifuged at 100,000 × *g* for 1 h. The supernatant containing the target protein was collected and incubated with Ni^2+^-NTA agarose resin (QIAGEN) for 2 h. The resin was sequentially washed by buffer A containing 10 mM Imidazole and buffer B (25 mM Tris–HCl, pH 7.5, 10% glycerol, 0.01% Igepal-CA-630, and 300 mM KCl) containing 20 mM Imidazole. HELQ protein was eluted with buffer B supplemented with 200 mM Imidazole. The elution was then added to anti-Flag M2 affinity agarose (Sigma–Aldrich). Then, the resin was washed with buffer B, and HELQ protein was eluted with buffer B supplemented with 3× Flag peptide (100 μg/ml). Finally, the eluted protein was concentrated for Superdex 200 Increase 10/300 GL-based fractionation equilibrated with buffer C (25 mM Tris–HCl, pH 7.5, 10% glycerol, 0.01% Igepal-CA-630, 1 mM β-mercaptoethanol, and 0.5 mM EDTA) supplemented with 300 mM KCl. The protein was then fractioned, concentrated, and stored at −80°C. The HELQ K365M mutant was purified using the same protocol as the wild-type counterpart. To prepare untagged HELQ for affinity pulldown experiments, 140 µg of (His)_6_-Flag-HELQ was incubated with 9 µg of His-tagged Tobacco etch virus (TEV) protease in Buffer C at 4°C overnight. To remove the TEV protease, the mixture, supplemented with 10 mM imidazole, was incubated with Ni-NTA agarose resin at 4°C for 2 h. Untagged HELQ was then collected from the flow-through of the Ni-NTA resin, concentrated, and stored at −80°C. The efficiency of tag removal was verified by western blot analysis using an anti-Flag antibody.

### Other recombinant proteins

Human RPA, RAD51, and RAD51 mutant variants were expressed in *Escherichia coli* and purified using previously described methods [8, 31, 32]. The BCDX2, BC, DX2, and MRE11 proteins were expressed in human Expi293F cells and purified according to established protocols [8].

### DNA substrates

The DNA substrates used in the fork reversal assays were designed from the previous report [33] and the oligonucleotides used are listed in the Supplementary Table S1. The fluorescence-labeled DNA oligonucleotides were purchased from Genomics, and the non-modified or phosphorothioate bond-modified DNA oligonucleotides were purchased from Integrated DNA Technologies. To prepare the leading strand gap DNA substrates for the fork reversal assay, oligo RF-A and RF-sB were first annealed in a molar ratio of 1:1.2 in the annealing buffer (50 mM Tris–HCl, pH 7.5, 10 mM MgCl_2_, 100 mM NaCl, and 1 mM DTT) to compose the upper strand of the fork substrates. In parallel, oligo RF-D and RF-C were annealed to form the bottom strand of the fork substrates. The mixture was heated at 80°C for 3 min, then moved to 65°C for 30 min, and cooled down to room temperature. The upper and bottom strands were mixed with an equal molar ratio and incubated at 37°C for 30 min. The annealed products were separated and purified from a 6% TBE–PAGE by electro-elution. The product was concentrated with an Amicon Ultra-4 concentrator (Millipore, NMWL 30 kDa) by centrifugation at 7000 × *g*, 4°C, and finally stored in the TE buffer (10 mM Tris–HCl, pH 8.0, and 0.5 mM EDTA). The lagging-strand gap substrates composed of RF-A, RF-B, RF-sC, and RF-D were annealed with the same procedure as the leading-strand gap substrate. The preparation of the 5′ overhang substrate for the fork protection assays was constructed as described in the previous report [8].

### Affinity pulldowns

The BCDX2 complex with His-XRCC2 (2 μg), BC with His-RAD51C, and DX2 with His-XRCC2 were first bound to the Ni-NTA beads (20 μl slurry) by incubating in 10 μl reaction buffer (35 mM Tris–HCl, pH 7.5, 10% glycerol, 0.01% Igepal-CA-630, 75 mM KCl, 1 mM DTT, and 30 mM imidazole) for 20 min at 37°C with a 1200 rpm shaker. After removing the unbound BCDX2/BC/DX2 from the Ni-NTA beads, 10 μl reaction buffer containing untagged HELQ (1.5 μg) was added to the beads and incubated at 37°C for an additional 40 min with a 1200 rpm shaker. The beads were then washed two times with 50 μl of reaction buffer. The supernatant portion was added with 4 μl of SDS sample buffer (0.2 M Tris–HCl, 0.28 M SDS, 40% glycerol, 2.86 M β-mercaptoethanol, and 1 mM bromophenol blue), and the beads were added with 12 μl of SDS sample buffer (0.1 M Tris–HCl, 0.14 M SDS, 20% glycerol, 1.43 M β-mercaptoethanol, and 0.5 mM bromophenol blue) before being analyzed by 10% sodium dodecyl–sulfate polyacrylamide gel electrophoresis (SDS–PAGE).

### TFO-directed DNA interstrand crosslink formation and mutagenesis assay

TFO, AG30 containing a covalent 5′ HMT-psoralen (pso-AG30) moiety, was synthesized and HPLC-purified by Midland Certified Reagent Co. (Midland, TX, USA) as previously described [34]. For mutagenesis assays, equimolar amounts of pso-AG30 and mutation-reporter plasmid pSupFG1, harboring a TFO binding site within the *supF* gene, were incubated in an amber tube with triplex binding buffer [50% glycerol, 10 mM Tris (pH 7.6), 10 mM MgCl_2_] at 37°C overnight, followed by 1.8 J/cm^2^ UVA (365 nm) irradiation on ice under a Mylar filter. The TFO-binding site is located within the *supF* gene in the reporter plasmid adjacent to a 5′-AT-3′ psoralen crosslinking site at the triplex–duplex junction. To confirm and quantify triplex-directed ICL formation, plasmids were linearized by EcoRI digestion, heat denatured, and resolved on a 1% alkaline agarose gel, stained with SYBR Gold, and visualized using a BIORAD Chemidoc imaging system. Densitometric quantification of band intensities was performed using ImageQuant software (GE Healthcare Life Sciences). To determine the effect of HELQ on processing TFO-directed ICLs in human cells, 2 μg of pSupFG1 with or without TFO-directed ICLs was transfected into HELQ-proficient or -deficient U2OS cells. Mutagenesis assays were performed as we have described [34]. In brief, cells were harvested 48 h after the plasmid transfection and plasmid DNA was isolated using the QIAprep Spin Miniprep kit (QIAGEN, Inc.). To remove unreplicated plasmids from the analysis, plasmids were treated with DpnI restriction endonuclease for 1 h at 37°C followed by phenol/chloroform/isoamyl alcohol extraction and ethanol precipitation with 0.3 M sodium acetate (pH 5.2). Subsequently, the plasmids were re-suspended in 10 μl nuclease-free water. Mutations in the supF gene due to processing of the TFO-directed ICLs were detected by transforming 30 μl of electro-competent *E. coli* cells MBM7070 (Lucigen) with 1 μl of plasmid DNA and then plating on X-gal, IPTG, and ampicillin-containing agar plates for blue/white screening. A functional supF gene produces a blue colony, while a mutated supF gene produces a white colony. Mutation frequencies were calculated by dividing the number of white colonies counted by the total number of colonies (blue + white) counted. Three individual experiments were performed for each mutagenesis assay and ∼15 000 colonies were counted per experiment. The *P*-values were calculated by the Holm–Sidak one-way analysis of variance method using Sigmaplot. Mutation spectra were determined by direct sequencing of the plasmid DNA.

## Results

### HELQ promotes fork slowing at stalled replication forks

Given HELQ’s biochemical activity on model stalled forks and its interaction with the RAD51 paralog complex BCDX2, we hypothesized that HELQ directly regulates replication fork dynamics under replication stress. To test this, we used DNA fiber assays to examine how HELQ loss affects drug-induced fork slowing.

To establish DNA damage conditions suitable for our DNA fiber analysis, we first monitored checkpoint activation by measuring phosphorylation of CHK1, CHK2, and ATM. Based on regimens used in prior studies [5, 10, 35, 36], we titrated multiple DNA-damaging agents to identify conditions that robustly induced replication stress while minimizing DSB formation. For the cell-based fork slowing analyses, we used MMC as our primary replication-stress model, given that HELQ deficiency confers pronounced sensitivity to ICLs [24–26]. CPT was included mainly as a short-term treatment for comparison and for the EM experiments. Phosphorylation of CHK1 at Ser345 (pCHK1) was used as a readout of replication stress, whereas phosphorylation of CHK2 at Thr68 (pCHK2) served as an indicator of DSBs; thus, conditions that produced strong pCHK1 but low pCHK2 were considered optimal for assessing fork remodeling. ATM phosphorylation at Ser1981 provided an additional measure of DSB signaling (Supplementary Fig. S1). Under our conditions, 100 nM and 1 µM CPT produced comparable levels of pCHK2 and pATM, while both induced weaker pCHK1 than HU, consistent with the idea that CPT primarily affects forks that encounter trapped TOP1 complexes [37]. Using these criteria, we selected working concentrations of MMC, CPT, and HU for subsequent DNA fiber assays.

**Figure 1.**
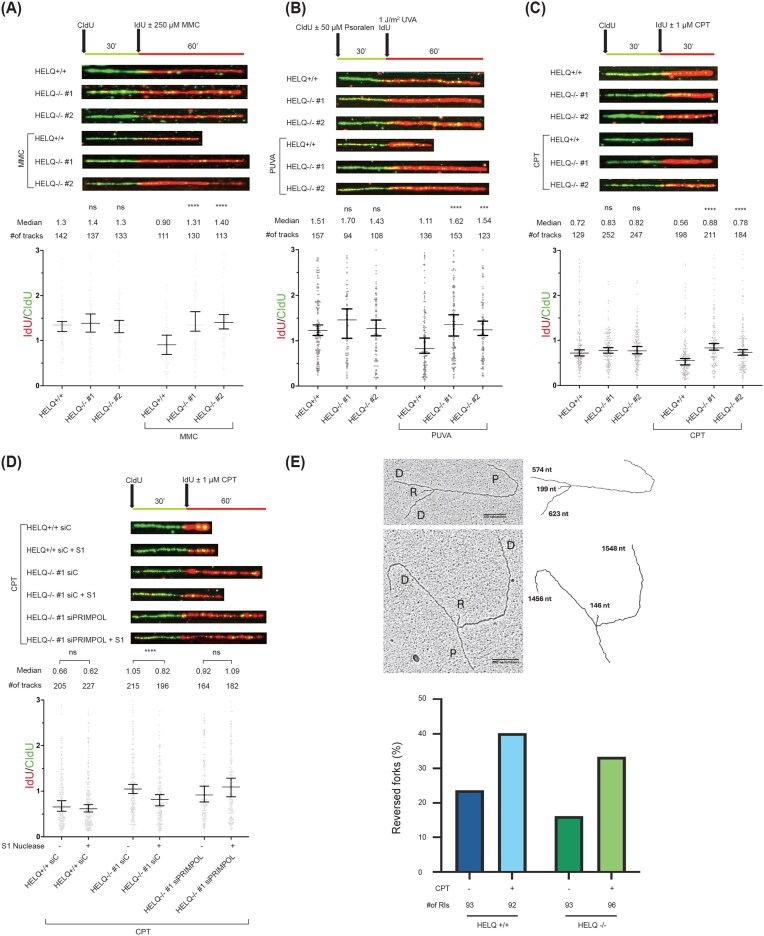
HELQ drives replication fork slowing by promoting fork reversal under replication stress. DNA fiber assays were performed in HELQ-proficient U2OS cells or HELQ-deficient U2OS clones (HELQ^−/−^ #1 and #2) labeled with chlorodeoxyuridine (CldU, green) for 30 min. Then cells were washed and incubated with iododeoxyuridine (IdU, red) together with or without (**A**) 250 µM mitomycin C (MMC) or (**C**) 1 µM camptothecin (CPT) for the indicated time before DNA combing. (**B**) For psoralen + UVA (PUVA), cells were first treated with CldU together with 50 µM psoralen and then subjected to 1 J/m^2^ UVA irradiation after a washing step for 15 min before being treated with IdU. (**D**) PRIMPOL was downregulated using siRNA transfection, and fork progression assay was performed as above, except for addition of S1 nuclease digestion step after DNA damaging agent pulse. The ratio between red and green tracts is plotted. The labeling scheme of the DNA fiber experiments and representative DNA fibers from each genetic condition are shown above each graph. At least 100 tracks were measured per condition in each of three independent experiments, which yielded similar results. Kruskal–Wallis test was used to assess statistical significance: ns not significant; ****P-value < .0001, ***P-value < .001, ** *P*-value <.01. (**E**) Ratios of reversed forks in HELQ-proficient and -deficient U2OS cells. G1/S synchronized cells were treated with 1 µM CPT or DMSO for 30 minutes and then processed for transmission electron microscopy. Quantities of replication intermediates (RIs) counted for each treatment are shown below each group. P = parental strand; D = daughter strand; R = reversed arm. Representative TEM image of reversed replication intermediates is shown above.

Since the 3′–5′ DNA helicase activity of HELQ can unwind DNA substrates mimicking stalled replication forks *in vitro* [22, 23], we examined fork progression under replication stress in HELQ knockout cell lines [25]. HELQ-proficient and HELQ-deficient cells were sequentially labeled with chlorodeoxyuridine (CldU) and iododeoxyuridine (IdU) in the presence or absence of drug-induced DNA damage, and replication fork progression was monitored by DNA fiber assays (Fig. 1 and Supplementary Fig. S2). Under unchallenged conditions, wild-type and HELQ-deficient cells exhibited comparable fork speeds. Upon treatment with ICL-inducing agents such as mitomycin C (MMC) and psoralen plus UVA (PUVA) (Fig. 1A and B) as well as a non-ICL-inducing agent, camptothecin (CPT) (Fig. 1C), wild-type cells displayed fork slowing, reflected by a reduction in IdU/CldU ratios. In contrast, this drug-induced fork slowdown was markedly attenuated in two independent HELQ-knockout clones (Fig. 1 and Supplementary Fig. S2A–C). Consistent with these conditions inducing replication stress, increased levels of CHK1 phosphorylation, a marker of DNA replication inhibition, were observed in parallel (Supplementary Fig. S1). These trends were similar when SMARCAL1, a well-established fork reversal factor, was depleted in parallel via siRNA knockdown (Supplementary Fig. S5A).

In the absence of fork reversal, cells are thought to rely more heavily on alternative, potentially error-prone DNA damage tolerance pathways such as translesion synthesis (TLS) and PRIMPOL-mediated lesion bypass. To further test HELQ’s involvement in fork reversal, PRIMPOL was transiently downregulated in HELQ-proficient and HELQ-deficient cells, and an S1 nuclease assay was performed to detect ssDNA gaps formed during replication (Fig. 1D and Supplementary Fig. S2D). In the absence of HELQ, cells relied more heavily on DNA damage tolerance pathways that generate single-stranded DNA gaps, a substrate for S1 nuclease digestion. This phenotype was similar to that observed upon siRNA-mediated downregulation of other established fork remodeling enzymes [6, 38, 39]. Together, these results support a model in which HELQ promotes fork remodeling and restrains a compensatory shift toward PRIMPOL-dependent gap-forming tolerance pathways.

For more direct confirmation, we visualized forks by transmission electron microscopy, an established approach for detecting replication intermediates (RIs) [6, 29, 40]. HELQ-deficient cells exhibited a modest reduction in the proportion of reversed forks (Fig. 1E). We interpret this EM dataset as direct structural validation that our treatment conditions generate reversed fork intermediates and that HELQ contributes to their formation.

### HELQ ATPase and helicase activities are required to promote fork reversal

**Figure 2.**
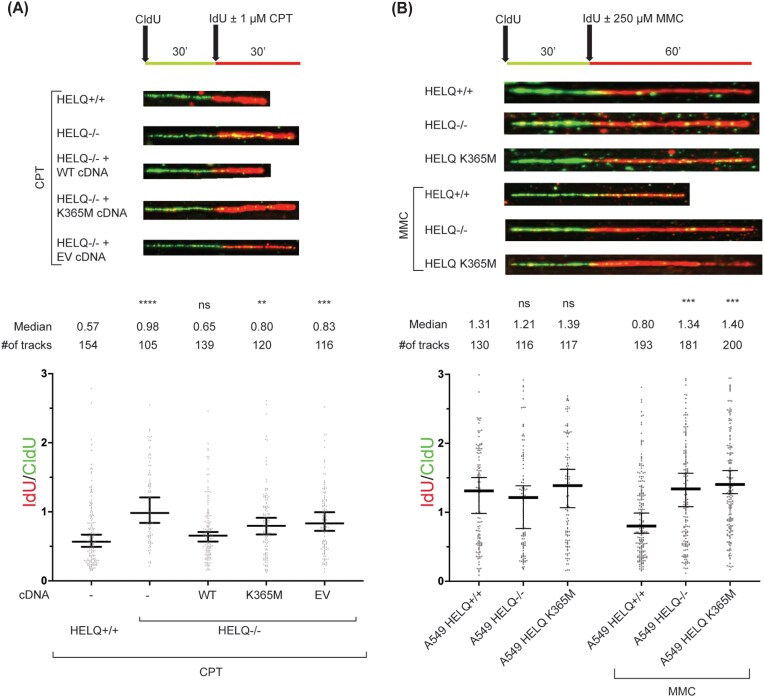
HELQ-mediated replication fork slowing is dependent on its ATPase and helicase activities. (**A**) DNA fiber assays were performed in HELQ-proficient U2OS cells or HELQ-deficient U2OS clone (HELQ^−/−^ #2) stably complemented with pOZ FH N HELQ WT or ATPase-dead K365M mutant. Cells were treated with 1 µM CPT before proceeding with DNA combing as described. (**B**) Lung cancer A549 cells proficient in HELQ, CRISPR–Cas9 biallelic knockout of HELQ, or with CRISPR–Cas9-introduced K365M mutation were treated with 250 µM MMC for the indicated time before DNA combing. The ratio between red and green tracts is plotted. The labeling scheme of the DNA fiber experiments and representative DNA fibers from each genetic condition are shown above each graph. At least 100 tracks were measured per condition in each of three independent experiments, which yielded similar results. Kruskal–Wallis test was used to assess statistical significance: ns not significant; ****P-value < .0001, ***P-value < .001, ** *P*-value <.01.

To determine whether the catalytic activity of HELQ is required for fork remodeling, we first performed complementation experiments in HELQ-deficient U2OS cells. Expression of wild-type HELQ cDNA restored CPT-induced fork slowing to levels comparable to HELQ-proficient controls, confirming that the fork slowing defect observed in HELQ knockout cells (Fig. 1) is specifically attributable to HELQ loss (Fig. 2A and Supplementary Fig. S3A). In contrast, expression of an ATPase-defective HELQ mutant (K365M), which abolishes helicase activity [21], failed to rescue the fork slowing defect and behaved similarly to the empty-vector control, indicating that HELQ ATPase/helicase activity is essential for efficient fork deceleration upon DNA damage (Fig. 2A and Supplementary Fig. S3A).

To exclude the possibility that this phenotype is restricted to U2OS cells, we generated isogenic HELQ knockout and HELQ K365M knock-in A549 cell lines and analyzed fork progression under MMC-induced replication stress. As in U2OS cells, HELQ loss in A549 cells attenuated fork slowing, and introduction of the K365M mutation in the endogenous HELQ locus phenocopied the knockout, abolishing MMC-induced fork slowdown (Fig. 2B and Supplementary Fig. S3B). Together, these results demonstrate that the ATPase/helicase activity of HELQ is required to promote replication fork slowing and, by extension, fork reversal in response to DNA damage.

### Depletion of BCDX2 does not further alter fork speed in HELQ-deficient cells under replication stress

**Figure 3.**
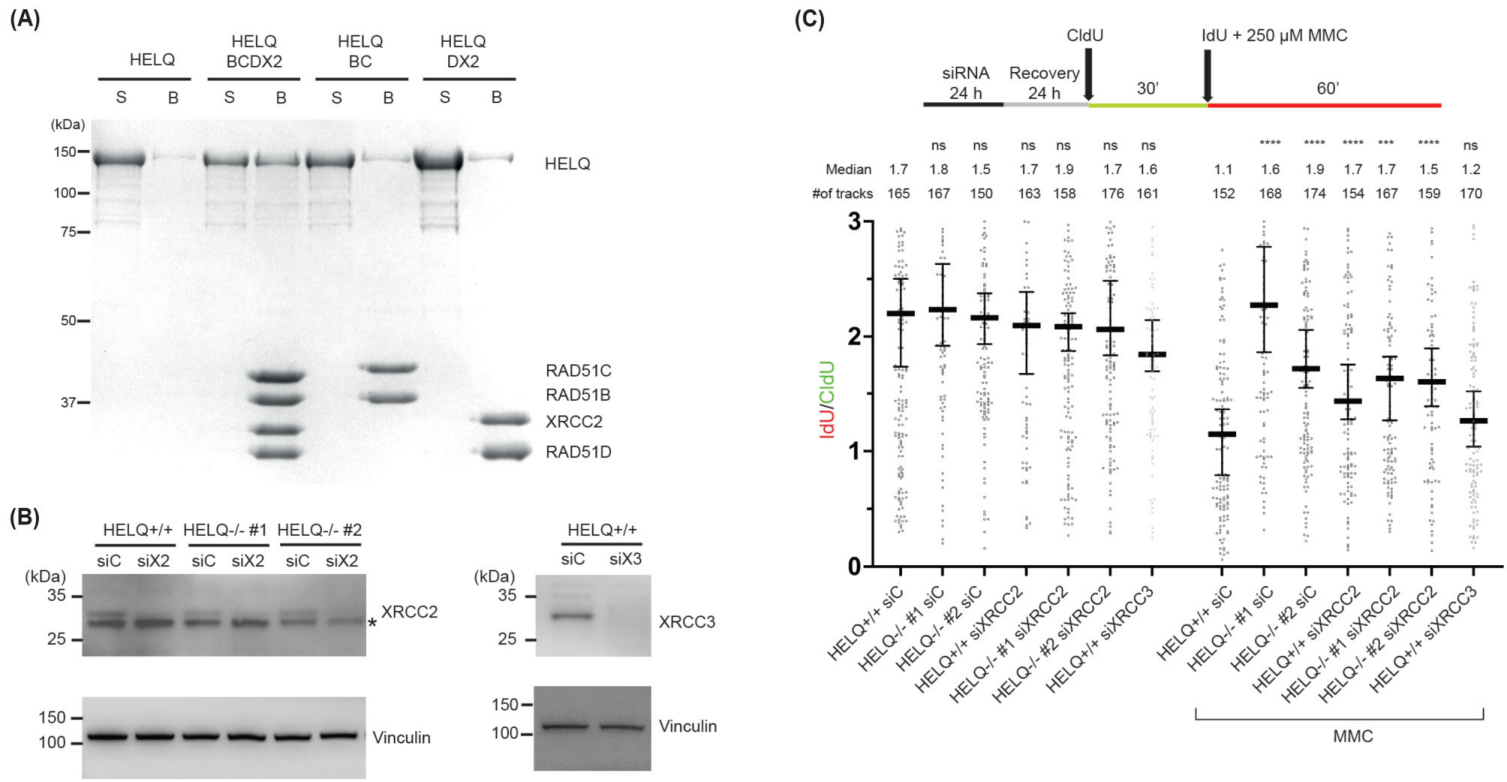
The epistatic relationship between HELQ and XRCC2 in replication fork slowing. (**A**) Protein interaction analysis of BCDX2 with His-XRCC2, BC with His-RAD51C, DX2 with His-XRCC2, and HELQ using Ni-NTA pull-down assay. The supernatant (S) and SDS elute (B) from the pull-down reactions were analyzed by 10% SDS–PAGE gel electrophoresis and detected by Coomassie Blue staining. (**B**) Immunoblotting analysis of RAD51 paralogs XRCC2 and XRCC3 upon 24 h downregulation with siRNA in HELQ-proficient and -deficient U2OS cells. siC, siX2, and siX3 indicate control siRNA, siXRCC2, and siXRCC3, respectively. * indicates non-specific bands. (**C**) The cells were sequentially labeled with CldU and IdU together with 250 µM MMC for the indicated time before DNA combing. Median values for IdU/CldU track ratios (from >150 tracks) are represented by lines. The experiment was performed twice, yielding similar results. Statistical analysis: Mann Whitney test; n.s., not significant; **** *P*-value <.0001; *** *P*-value <.001; ** *P*-value <.01.

It has been reported that HELQ co-immunoprecipitates with the BCDX2 complex, but not with X3CDX2, from cellular extract [25, 26]. This observation raises the question of whether HELQ and BCDX2 engage in functional crosstalk during the replication-stress response. To further examine this interaction, we conducted *in vitro* pull-down assays using purified proteins. Consistent with a previous study [26], the purified BCDX2 directly bound HELQ (Fig. 3A). Notably, the intact BCDX2 complex displayed a higher affinity for HELQ than either the BC or DX2 subcomplex alone (Fig. 3A).

Because BCDX2 promotes fork slowing, whereas X3CDX2 functions in fork restart [5, 11], we asked whether HELQ acts together with BCDX2 to slow replication forks. We depleted XRCC2 (a BCDX2 subunit) in parental U2OS cells and in two independent HELQ-knockout U2OS clones, and, in parallel, depleted XRCC3 (an X3CDX2 subunit) in U2OS cells (Fig. 3B and Supplementary Fig. S4). We then monitored fork progression under MMC-induced replication stress (Fig. 3C and Supplementary Fig. S4). Consistent with prior work [5], XRCC2 depletion—but not XRCC3 depletion—relieved MMC-induced fork slowing in U2OS cells, restoring fork speed (Fig. 3C and Supplementary Fig. S4). In line with our model, co-depletion of HELQ and XRCC2 did not produce an additional increase in fork speed beyond HELQ loss alone, indicating that HELQ is epistatic to BCDX2 for replication fork slowing.

### HELQ possesses ATP-dependent fork regression activity and is stimulated by RPA on leading strand gap fork substrates

**Figure 4.**
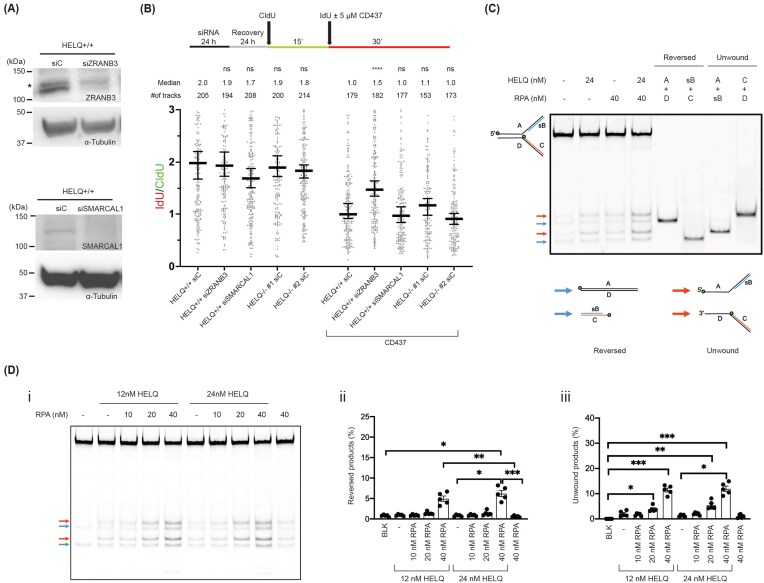
HELQ is stimulated by RPA on leading strand gap substrates, and Pol α inhibition does not identify a major role for HELQ in this cellular context. (**A**) Immunoblotting analysis of ZRANB3 and SMARCAL1 following 24 h downregulation with siRNA in HELQ-proficient U2OS cells. * indicates non-specific bands. (**B**) Fork remodeling assays were performed in U2OS cells treated with the Pol α inhibitor CD437. Under these conditions, HELQ knockout behaved similarly to SMARCAL1 depletion and did not restore fork speed. The experiment was performed three times, yielding similar results. At least 100 tracks were measured for each set. (**C**) RPA stimulates HELQ’s helicase activity and increases both reversed and unwound products with leading strand gap substrates. The sizes of the reversed and unwound products are shown in the gel. The schematic diagram shows the reversed (blue arrows) and the unwound product (red arrows), respectively. (**D**) Twelve and twenty-four nM of HELQ were incubated with various concentrations of RPA in the reaction buffer, then leading strand gap DNA substrate (16 nM) was added to start the reaction for a further 20 min. Blue and red arrows indicate reversed and unwound products, respectively. (i) Representative experiment. (ii) Quantification of reversed products (error bars show SEM of five replicates). (iii) Quantification of unwinding products (error bars show SEM of five replicates). Statistical significance was determined using one-way ANOVA with the Kruskal–Wallis test; ns not significant, *P < .05, **P < .01, ***P < .001, ****P < .0001.

Replication fork stalling can be triggered by DNA damage on the leading strand, the lagging strand, or both strands (e.g. an ICL). Multiple fork reversal enzymes act on distinct classes of stalled forks. For example, when lagging strand priming was inhibited by a Pol α inhibitor (CD437), RAD51, ZRANB3, and HLTF cooperated to promote fork slowing, whereas SMARCAL1 was dispensable [41]. Consistent with this, depletion of ZRANB3, but not SMARCAL1, restored fork speed in CD437-treated cells (Fig. 4A and B and Supplementary Fig. S5B). In the same assay, HELQ knockout also failed to rescue fork speed, similar to SMARCAL1 depletion. Thus, under our conditions, HELQ is not a major determinant of the fork remodeling response triggered by Pol α inhibition. We do not interpret this cellular assay as direct evidence that HELQ acts specifically at sites of leading strand blockage *in vivo*. Instead, support for the use of leading strand gap substrates comes from the biochemical reconstitution experiments described below, which employ defined fork substrates.

To this end, we expressed and purified HELQ from human Expi293 cells (Supplementary Fig. S6A). We used several annealed model fork substrates, including forks with leading and lagging strand gaps, and incubated them with HELQ at various concentrations and incubation times [33] (Supplementary Fig. S6B, schematic diagram). Interestingly, no evident fork reversal activity by HELQ was observed when a leading strand gap substrate was utilized in a reconstituted model fork reversal assay (Supplementary Fig. S6C-i). Notably, RPA has been shown to enhance HELQ helicase activity [21]. We therefore investigated whether RPA could enhance HELQ’s fork reversal activity on the leading strand gap substrate. Upon addition of purified human RPA together with HELQ, we observed a clear RPA-dependent increase in both reversed (blue arrows) and unwound (red arrows) products (Fig. 4C and D). In this setting, the ssDNA gap on the leading strand served as a binding site for both RPA and HELQ; upon binding, HELQ unwound the parental strands with its characteristic 3′–5′ polarity, generating two unwound products with 3′ and 5′ overhangs (Fig. 4D-iii). Partial separation of the parental duplex further exposes ssDNA on the lagging strand, resulting in the formation of a minor population of reversed fork products (Fig. 4D-ii). These findings are consistent with previous studies and underscore the stimulatory role of RPA in HELQ-mediated fork remodeling [22, 23].

Because HELQ is a 3′–5′ helicase, it readily catalyzed fork reversal on the substrate bearing a lagging strand ssDNA gap (Supplementary Fig. S6C-ii and D). Notably, such a lagging strand gap substrate can be generated from the leading strand gap substrate following HELQ- and RPA-dependent unwinding of the parental duplex (Fig. 4C), providing a mechanistic link between these reactions. To determine whether HELQ’s fork remodeling activity stems from its helicase function, we included the HELQ K365M mutant, which lacks helicase activity [21], in our functional analysis. As expected, HELQ K365M produced no detectable fork reversal, even in the presence of ATP (Supplementary Fig. S6E, lanes 8–10). Thus, HELQ is a *bona fide* ATP-dependent fork remodeler, and its ATPase/helicase activity is required for fork reversal *in vitro*.

Next, we examined whether HELQ–BCDX2 interaction influences HELQ-mediated fork reversal activity. Various concentrations of BCDX2 were included in the HELQ-mediated fork reversal assay using both leading and lagging strand gap substrates. Under these conditions, addition of BCDX2 neither stimulated nor inhibited HELQ-mediated fork reversal (Supplementary Fig. S6F and G), suggesting that the HELQ–BCDX2 interaction may support other aspects of HELQ function in cells, or that additional factors and/or substrate contexts are required to reveal its impact on fork reversal in our reconstituted system.

### The fork remodeling activity of HELQ promotes MRE11-dependent fork degradation in the absence of BRCA2 or FANCD2

BRCA2 and FANCD2 are important to protect reversed forks from nucleolytic degradation [17, 42]. Deficiency of BRCA2 or FANCD2 promotes fork degradation, which is alleviated by inhibition of MRE11 [7, 9]. Here, we show that HELQ promotes fork slowing upon DNA damage and catalyzes fork reversal *in vitro* (this work). If HELQ contributes to fork reversal in cells, then loss of HELQ should suppress fork degradation in BRCA2- or FANCD2-deficient backgrounds, because fork protection is primarily required when forks undergo reversal.

**Figure 5.**
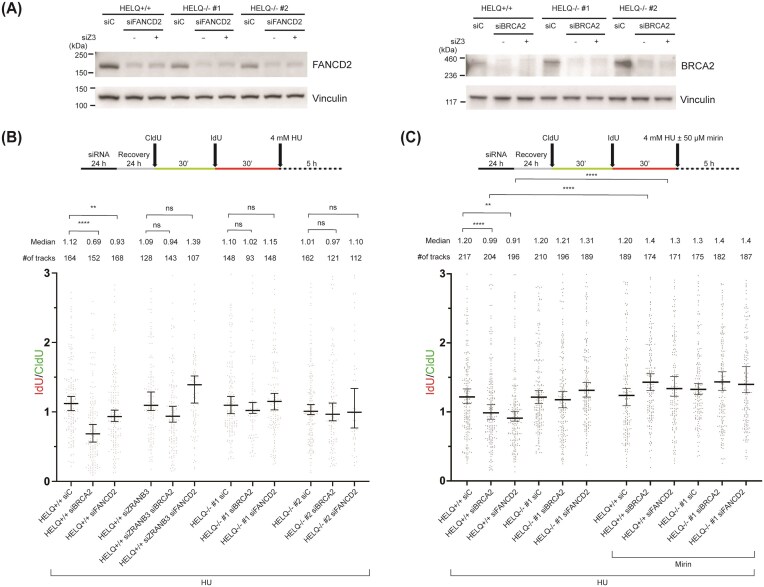
Restoration of replication fork stability in BRCA2- and FANCD2-deficient cells by HELQ knockout. (**A**) Immunoblotting analysis of BRCA2 and FANCD2 following 24 h downregulation with siRNA in HELQ-proficient and -deficient U2OS cells. (**B**) 24 h upon siBRCA2, siFANCD2 or siZRANB3 treatment cells were sequentially incubated with CldU for 30 min, IdU for 30 min, and 4 mM HU for 5 h before DNA combing. Median values for IdU/CldU track ratios (from >100 tracks) are represented by lines. The experiment was performed three times, yielding similar results. ZRANB3, which promotes fork remodeling, was knocked down in U2OS cells and used as control. (**C**) Same as in panel (B) except cells were treated with 50 µM mirin concomitantly with HU. siC, siBR2, and siFD2 indicate control siRNA, siBRCA2, and siFANCD2, respectively. Kruskal–Wallis test was used to assess statistical significance: n.s., not significant; **** *P*-value <.0001; *** *P*-value <.001; ** *P*-value <.01.

To test this prediction, we depleted BRCA2 or FANCD2 in HELQ^−/−^ cells (Fig. 5A) and monitored nascent strand degradation by DNA fiber analysis (Fig. 5B and C and Supplementary Fig. S7A and B). Consistent with previously published results [7, 9], depletion of BRCA2 or FANCD2 promoted fork degradation upon HU treatment (Fig. 5B and Supplementary Fig. S7). Importantly, HELQ deletion rescued fork degradation in BRCA2- or FANCD2-depleted cells, supporting a model in which HELQ-dependent fork remodeling generates the reversed fork substrates that require BRCA2/FANCD2-mediated protection. As control, we examined ZRANB3, an established fork reversal factor [42]. Consistent with previous work, ZRANB3 depletion suppressed fork degradation in BRCA2- or FANCD2-deficient cells (Supplementary Fig. S7).

We further confirmed that the degradation observed in BRCA2- or FANCD2-depleted cells was MRE11-dependent using the MRE11 inhibitor mirin. Mirin treatment rescued HU-induced fork degradation in BRCA2- or FANCD2-depleted cells. In contrast, mirin had little to no additional effect when HELQ was absent, consistent with reduced fork reversal and therefore reduced generation of MRE11-sensitive reversed fork substrates in HELQ-deficient cells (Fig. 5C and Supplementary Fig. S7C).

### HELQ enhances RAD51-mediated fork protection against MRE11-dependent degradation

**Figure 6.**
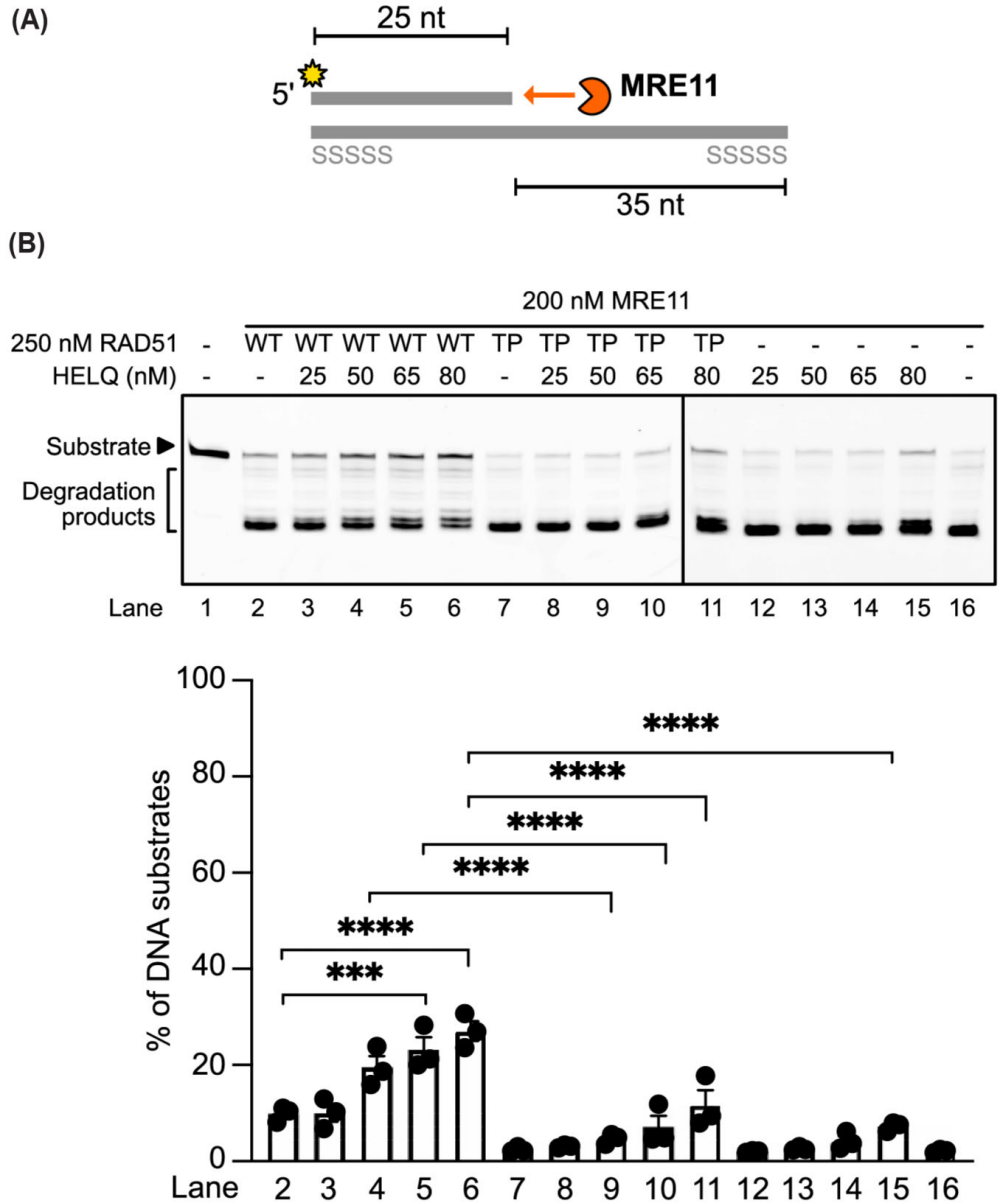
HELQ facilitates RAD51 fork protection against MRE11 degradation. (**A**) Illustration of the protection assay with 5′ overhang substrates mimicking the regressed arm of the reversed fork. The 5′ overhang substrate was assembled using a 5′ Cy3-labeled 25-nucleotide (nt) oligonucleotide and a 60 nt oligonucleotide with a phosphorothioate bond modification on both ends to limit the digestion direction by MRE11. (**B**) HELQ synergizes with wild-type RAD51 but not the DNA-binding defective mutant (T131P). HELQ at the indicated concentration and RAD51 wild type or T131P (TP) were first incubated with DNA substrates (29 nM) at 37°C for 10 min, and then MRE11 was added for a further 40-min digestion. (Top) a representative gel image (Bottom) quantitative data (error bars show SEM of three replicates). Statistical significance was determined using one-way ANOVA with Tukey’s post hoc test; ns not significant, *P < .05, **P < .01, ***P < .001, ****P < .0001.

Our cellular experiments support a role for HELQ in fork reversal. However, these results do not exclude an additional function for HELQ in fork protection. Because HELQ-knockout cells form fewer reversed forks in DNA fiber analyses, fork protection is difficult to assess in that setting, as the substrates that require protection are reduced. Notably, HELQ’s ssDNA-binding activity has been proposed to protect stalled forks from nucleolytic attack, including MRE11 [43]. Given the central requirement for RAD51 in fork protection, we designed experiments to test whether HELQ potentiates RAD51-dependent protection of stalled forks (Fig. 6A).

In this assay, HELQ alone provided only modest protection against MRE11-mediated degradation. By contrast, inclusion of RAD51 revealed a strong cooperative effect between RAD51 and HELQ (Fig. 6B, compare lanes 3–6 to 12–15 and 2). To determine whether this cooperation depends on stable RAD51 filament formation, we tested the RAD51 T131P mutant, which is defective in DNA binding. Substituting RAD51 T131P markedly reduced the cooperative protection, compared with wild-type RAD51 (Fig. 6B, compare lanes 3–6 with lanes 8–11), indicating that stable RAD51 filament formation is required for HELQ-enhanced fork protection.

### Restoration of replication fork progression after DNA damage is not impaired in HELQ knockout cells

**Figure 7.**
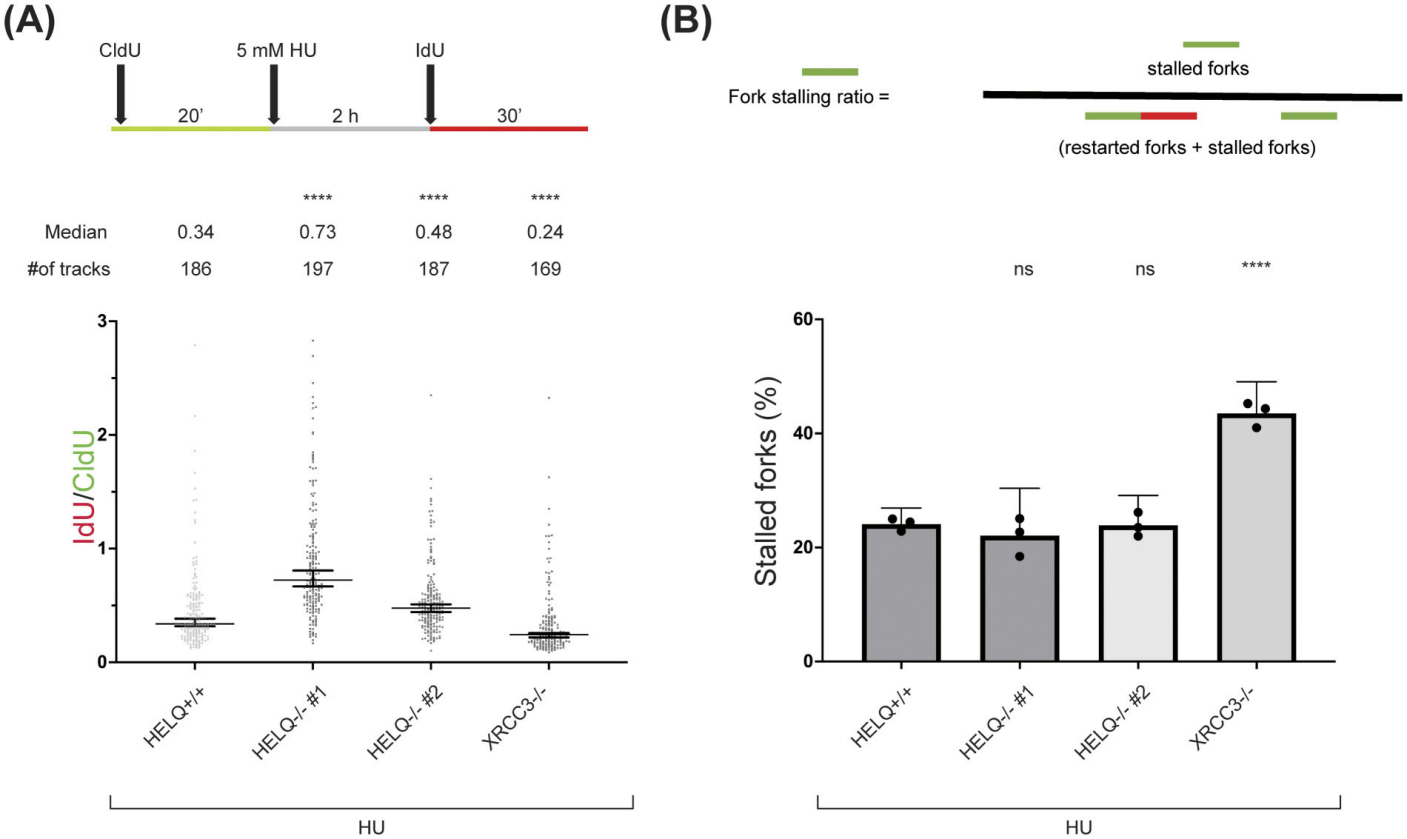
HELQ does not promote reversed fork restart in U2OS cells. (**A**) DNA fiber analysis of U2OS cells to investigate replication fork restart upon HU treatment. Top: schematic CldU/IdU pulse-labeling protocol to evaluate fork restart upon the treatment with HU (5 mM, 2 h). Median values for IdU/CldU track ratios (from >100 tracks) are represented by lines. (**B**) Fork stalling ratio was analyzed from the same images as in panel (A) (>300 tracks). The experiment was performed three times, yielding similar results. Kruskal–Wallis test was used to assess statistical significance in panel (A); one-way ANOVA with Bonferroni correction was used for panel (B): n.s., not significant; **** *P*-value <.0001; *** *P*-value <.001; ** *P*-value <.01.

We next tested whether HELQ contributes to restart of reversed replication forks by measuring nascent DNA synthesis after release from HU-induced stalling (Fig. 7A and Supplementary Fig. S8A). We also measured the fraction of forks that remained stalled after HU treatment (Fig. 7B). Consistent with a previous report [5], XRCC3 depletion reduced fork restart efficiency (Fig. 7A). In contrast, HELQ deletion did not impair fork restart and instead resulted in unchanged or slightly increased IdU/CldU ratios. This pattern resembles that reported for RAD51 or BCDX2, which promote fork reversal but are not required for fork restart [5, 6]. The modest increase in IdU/CldU ratios may reflect impaired fork reversal in HELQ-knockout cells, as non-reversed forks may resume DNA synthesis more readily, potentially via efficient repriming events [2, 5]. Together, these data indicate that HELQ is dispensable for replication fork restart under these conditions.

### HELQ limits deletion mutagenesis during ICL repair in human cells

**Figure 8.**
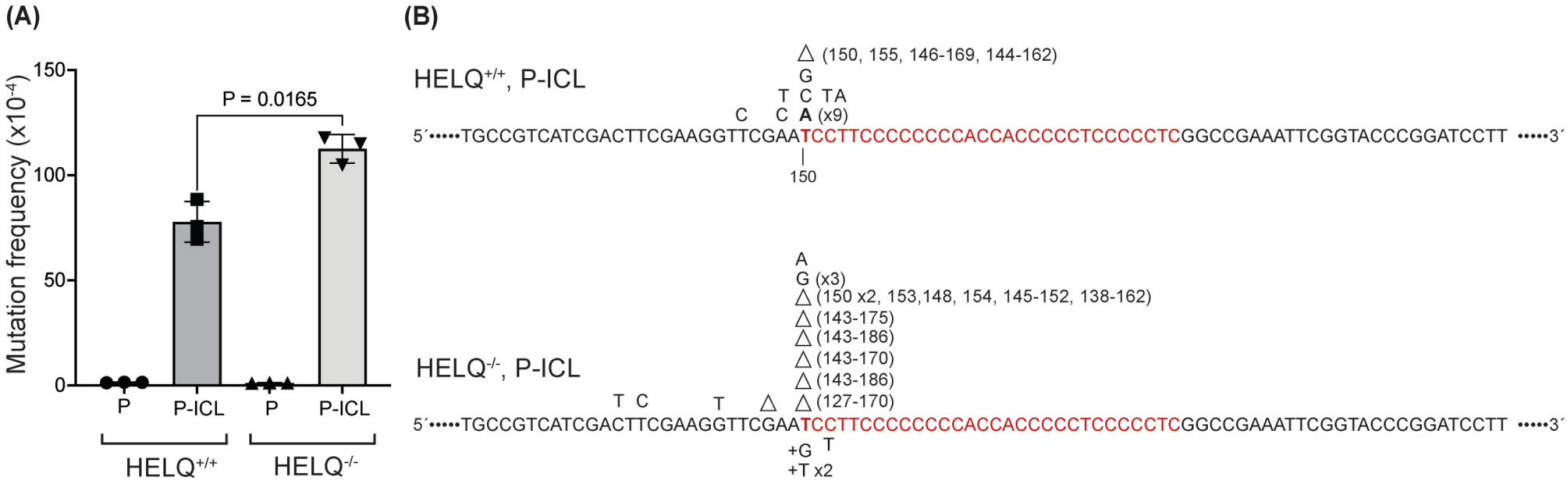
The absence of HELQ in human cells increases TFO-directed ICL-induced mutagenesis. (**A**) Spontaneous (P) and ICL-induced (P-ICL) mutation frequencies in HELQ-proficient and -deficient U2OS cells, respectively. Cells were transfected with the pSupFG1 mutation-reporter plasmid (P) or with TFO-directed psoralen-crosslinked pSupFG1 (P-ICL), as indicated under the bars. These data were generated from three rounds of transfection and counting ~15 000 colonies per experimental group; error bars represent ± SD. *P*-values derived from paired two-sample Student’s t-tests. (**B**) The mutation spectra generated while processing TFO-directed ICLs in U2OS cells. While the HELQ-proficient cells predominantly demonstrate T to A point mutation at the targeted T (highlighted in bold font and identified as residue 150), the HELQ-deficient cells demonstrate deletions at the targeted T site. A total of 20 mutants were sequenced from three independent experiments.

We tested whether HELQ limits mutagenic processing of ICLs in human cells using a triplex-forming oligonucleotide (TFO)-directed ICL mutagenesis assay with a replication-proficient donor plasmid carrying a wild-type *supF* gene [34]. HELQ loss increased ICL-induced mutagenesis by ∼1.5-fold in U2OS cells (Fig. 8A). To define the mutation spectrum, we isolated plasmid DNA from 20 randomly selected mutant colonies per condition and sequenced the region surrounding the TFO-targeted 5′-AT-3′ psoralen crosslink site. In HELQ-proficient cells, the predominant mutation class was T→A transversions at the targeted thymidine, consistent with previous work [34]. In contrast, in HELQ-deficient cells, deletions predominated at the targeted site (Fig. 8B), suggesting that HELQ-dependent fork remodeling suppresses error-prone outcomes—particularly deletions—during ICL processing. Fork reversal and PRIMPOL-mediated repriming (ICL traverse) represent two alternative strategies for dealing with ICLs during replication [20, 38]. In the absence of HELQ-mediated fork reversal, increased reliance on PRIMPOL-dependent repriming could bypass lesions and leave behind ssDNA gaps, which can subsequently be processed into deletions [44, 45].

## Discussion

DNA helicase HELQ is a 3′–5′ DNA helicase with recently discovered DNA strand annealing activity [46, 47] and is one of the three mammalian Mus308 homologs (HELQ, POLQ, and POLN) [21, 48]. In *Drosophila*, Mus308 is important for ICL resistance [49]. In mammalian cells, loss of HELQ increases sensitivity to ICL-forming agents such as cisplatin and mitomycin C [24–26], whereas loss of POLQ or POLN does not produce comparable hypersensitivity [48, 50–52]. Why HELQ deficiency specifically confers pronounced ICL sensitivity has remained unclear.

ICLs are extremely cytotoxic, but the mechanism of their repair is not fully understood. Studies with *Xenopus* egg extracts demonstrated that the repair of a cisplatin ICL is triggered when two replication forks converge on the lesion, generating an X-shaped structure [53]. However, this X-shaped intermediate is not an efficient substrate for XPF–ERCC1, which makes incisions on both the 5′ and 3′ sides of the ICL. Fork reversal can convert the X-shaped structure into a Y-shaped intermediate that supports XPF–ERCC1 incision and ICL unhooking [54, 55]. The fork reversal for ICL damage is therefore an important step to repair ICL, and it requires ubiquitin-dependent unfolding and unloading of the CDC45/MCM2-7/GINS (CMG) helicase by p97 [55]. In this context, RAD51 is dispensable for fork reversal when CMG is unloaded, whereas RAD51 promotes fork reversal at non-ICL lesions when CMG remains engaged [6]. Together, these findings indicate that different classes of stalled forks are processed by distinct fork reversal mechanisms.

Building on prior work showing that HELQ directly interacts with the RAD51 paralog complex BCDX2 [25, 26], which has been implicated in fork reversal as an early step in fork remodeling [5], we investigated whether HELQ functionally contributes to replication fork reversal under replication stress. Our cell-based assays, DNA fiber, transmission electron microscopy, and biochemical analyses indicate that HELQ promotes replication fork slowing and contributes to fork reversal in response to replication stress. Importantly, HELQ functions epistatically with XRCC2, a core BCDX2 subunit, in fork slowing. Additional support for a HELQ role in fork reversal comes from the observation that HELQ inactivation suppresses MRE11-dependent nascent strand degradation in BRCA2- or FANCD2-deficient cells (Fig. 5), mirroring genetic suppression seen upon perturbation of other fork remodelers such as SMARCAL1, ZRANB3, and HLTF [6, 10]. Moreover, DNA fiber experiments using the DNA polymerase α inhibitor CD437 suggest that HELQ preferentially acts at forks bearing leading strand template ssDNA, analogous to SMARCAL1, which is recruited via RPA-coated ssDNA on the leading strand (Fig. 4). Direct combinatorial experiments with SMARCAL1, ZRANB3, or HLTF will be important in future studies to determine whether HELQ functions independently, redundantly, or in concert with these canonical fork remodelers.

Consistent with these cellular findings, our reconstituted biochemical assays establish HELQ as a bona fide fork remodeler capable of catalyzing fork reversal. Given HELQ’s 3′–5′ helicase polarity, it is not surprising that HELQ more efficiently reverses substrates bearing a lagging strand ssDNA gap than those with a leading strand gap. Notably, we find that RPA strongly enhances HELQ-mediated fork reversal on leading strand gap substrates. Integrating the cellular and biochemical data, we propose a working model in which HELQ acts at forks containing a leading strand ssDNA gap (Supplementary Fig. S8B). In this model, HELQ, supported by RPA, initiates parental duplex unwinding from the leading strand gap, which in turn exposes ssDNA on the lagging strand and generates a substrate that HELQ can more efficiently convert into a reversed fork. This framework is compatible with reports that HELQ can functionally interact with RPA and displace it from ssDNA [22, 23], a property that could facilitate HELQ engagement at RPA-bound leading strand ssDNA and promote a transition toward RAD51-bound intermediates. In addition, HELQ’s DNA strand–annealing activity [46, 47] may help anneal nascent strands to form a four-way junction, potentially contributing to efficient formation or stabilization of reversed forks. The mechanistic significance of the HELQ–RPA axis in fork reversal will require further dissection.

Although HELQ-deficient cells do not show overt defects in the subsequent steps of fork remodeling, fork protection and restart, we do not exclude potential HELQ contributions to these processes. In our cellular system, reduced fork reversal in HELQ-knockout cells limits the availability of reversed-fork substrates, making it difficult to directly assess fork protection and restart. Nevertheless, our *in vitro* assays indicate that HELQ enhances RAD51-mediated fork protection against MRE11-dependent degradation. This cooperative protection may reflect physical or functional coupling between HELQ and RAD51, consistent with reports of a direct HELQ–RAD51 interaction [47] and reduced RAD51 recruitment in HELQ-deficient cells [43]. It will be important in future work to test a broader panel of informative HELQ mutants, including the ATPase/helicase-dead mutant K365M, to determine whether HELQ’s roles in fork protection and fork remodeling can be genetically uncoupled. In our system, K365M is clearly defective in fork remodeling, as it fails to rescue drug-induced fork slowing in cells and does not support fork reversal *in vitro*, whereas its contribution to fork protection remains to be tested directly. Additional mutants, including the PWI-like domain mutant D142A/F143A, which perturbs RPA-coupled loading [56], N-terminal mutants that disrupt Pol δ/POLD3 interaction [57], and the ssDNA-binding mutant K587A [43], should further help define how substrate engagement and accessory interactions contribute to fork remodeling.

Our findings provide mechanistic insight into HELQ-mediated fork remodeling, a process that is central to the cellular response to fork-blocking DNA lesions. HELQ loss sensitizes human cells to ICL-forming agents [24–26], fork reversal facilitates ICL unhooking via XPF–ERCC1 [55], and HELQ deficiency increases mutagenesis during ICL processing (Fig. 8), collectively suggesting that HELQ-dependent fork reversal is particularly important for limiting toxic and mutagenic outcomes of ICL damage. Supporting this idea, a time-resolved, mass spectrometry–based analysis of proteins recruited to forks stalled at ICLs in Xenopus egg extracts detected enrichment of HELQ at damaged forks immediately prior to XPF–ERCC1 recruitment [58]. Together, these observations raise the possibility that HELQ inhibition could augment the cytotoxicity of ICL-based chemotherapy, although therapeutic exploitation will require careful evaluation of context, specificity, and genome-wide consequences.

## Supplementary Material

gkag332_Supplemental_Files

## Data Availability

The authors confirm that all relevant data are present in the manuscript and its supplementary data. Materials in this study are available from the corresponding author upon reasonable request.
