# Supplementary material for: The DNA helicase HELQ promotes replication fork reversal in coordination with BRCA2- and FANCD2-mediated repair pathways
Source: Nucleic Acids Res. 2026 Apr 29;54(8):gkag332. doi: 10.1093/nar/gkag332 (PMC13125761; doi:10.1093/nar/gkag332)
Supplement: gkag332_Supplemental_Files [file gkag332_supplemental_files.zip › Supplementary Table 1.docx]

**Supplementary Table 1.** List of oligonucleotides used in fork reversal. Cy3: represents Cy3 fluorescent dye-labeled on the indicated oligonucleotide's 5’- or 3’- end. The bottom line labeled on the RF-A and RF-D indicated the two mismatched nucleotides preventing spontaneous fork-reversal formation.

| oligo | Length (nt) | Sequence (5’-3’) |
| --- | --- | --- |
| RF-A | 122 | Cy3-CGTGACTTGATGTTAACCCTAACCCTAAGATATCGCGTTATCAGAGTGTGAGGATACATGTAGGCAATTGCCACGTGTCTATCAGCTGAAGTTGTTCGCGACGTGCGATCGTCGCTGCGACG |
| RF-B | 82 | CGTCGCAGCGACGATCGCACGTCGCGAACAACTTCAGCTGATAGACACGTGGCAATTGCCTACATGTATCCTCACACTCTGA |
| RF-C | 82 | Cy3-TCAGAGTGTGAGGATACATGTAGGCAATTGCCACGTGTCTATCAGCTGAAGTTGTTCGCGACGTGCGATCGTCGCTGCGACG |
| RF-D | 122 | CGTCGCAGCGACGATCGCACGTCGCGAACAACTTCAGCTGATAGACACGTGGCAATTGCCTACATGTATCCTCACACTCTGAATACGCGATATCTTAGGGTTAGGGTTAACATCAAGTCACG |
| RF-sB | 52 | CGTCGCAGCGACGATCGCACGTCGCGAACAACTTCAGCTGATAGACACGTGG |
| RF-sC | 52 | Cy3-CCACGTGTCTATCAGCTGAAGTTGTTCGCGACGTGCGATCGTCGCTGCGACG |
