# Supplementary material for: The DNA helicase HELQ promotes replication fork reversal in coordination with BRCA2- and FANCD2-mediated repair pathways
Source: Nucleic Acids Res. 2026 Apr 29;54(8):gkag332. doi: 10.1093/nar/gkag332 (PMC13125761; doi:10.1093/nar/gkag332)
Supplement: gkag332_Supplemental_Files [file gkag332_supplemental_files.zip › Supplementary Data.docx]

**Supplementary Figures**

The DNA helicase HELQ promotes replication fork reversal in coordination with BRCA2- and FANCD2-mediated repair pathways.

Yerkin Dunbayev ^1&2*^, Yen-Ju Chen ^3*^, Lorenzo Sassi ^4&5^, EunA Lee ^1^, Jae Sun Ra ^1^, Moonjung Choi ^1^, Anirban Mukherjee ^6^, Karen M. Vasquez ^6^, Vincenzo Costanzo ^4&5^, Peter Chi ^3&7§^, and Kei-ichi Takata ^1&8§^

* The first two authors should be regarded as Joint First Authors.

1 Center for Genomic Integrity, Institute for Basic Science, Ulsan 44919, Republic of Korea. 2 Department of Biological Sciences, Ulsan National Institute of Science and Technology, Ulsan 44919, Republic of Korea. 3Institute of Biochemical Sciences, National Taiwan University, Taipei, Taiwan. 4 Department of Oncology and Hematology-Oncology, University of Milan, Via Adamello 16, 20139 Milan, Italy. 5 IFOM-ETS, The AIRC Institute of Molecular Oncology, Milan, Italy. 6 Division of Pharmacology and Toxicology, College of Pharmacy, The University of Texas at Austin, Dell Pediatric Research Institute, 1400 Barbara Jordan Boulevard, Austin, TX 78723, USA. 7 Institute of Biological Chemistry, Academia Sinica, Taipei, Taiwan. 8 Department of Biomedical Engineering, College of Information-Bio Convergence Engineering, Ulsan National Institute of Science and Technology, Ulsan 44919, Republic of Korea.

Correspondence:

Kei-ichi Takata, Ph.D.

Center for Genomic Integrity, Institute for Basic Science (IBS), Ulsan 44919, Republic of Korea

Phone: +82-52-217-5536

Fax: +82-52-217-5519

E-mail: [ktakata@ibs.re.kr](mailto:ktakata@ibs.re.kr)

Peter Chi, Ph.D.

Institute of Biochemical Sciences, National Taiwan University, Taipei, Taiwan.

Tel: +886-2-23665573

Fax: +886-2-23635038

E-mail: [peterhchi@ntu.edu.tw](mailto:peterhchi@ntu.edu.tw)

**Supplementary Figure 1.** Immunoblotting analysis of ATR-mediated phosphorylation of CHK1 (Ser345), CHK2 (Thr68), ATM, and ATM (Ser1981) following treatment with the indicated DNA damaging agents for the specified durations in HELQ-proficient U2OS cells. Vinculin, loading control. The experiment was performed three times with similar results.

**Supplementary Figure 2. HELQ drives replication fork slowing by promoting fork reversal under replication stress.** Superplots showing three biological replicates of DNA fiber assays following treatment with MMC **(A)**, PUVA **(B)**, CPT **(C)**, or PRIMPOL perturbation **(D)**, together with scatter plots of individual IdU and CldU tract lengths (um) corresponding to the representative plots in Figure 1. The CldU/IdU (green/red) tract-length ratio is plotted. The DNA fiber labeling scheme and representative fibers for each genotype are shown above each graph. At least 100 fibers were quantified per condition in each of three independent experiments, which produced similar results.

**Supplementary Figure 3. HELQ-mediated replication fork slowing is dependent on HELQ ATPase/helicase activities.** Superplots combining three biological replicates of the DNA fiber assays in Fig. 2 A and B. The IdU/CldU (red/green) ratio is plotted. The DNA fiber labeling scheme is shown above each graph. At least 100 tracks were measured per condition in each of three independent experiments, which produced similar results.

**Supplementary Figure 4. The epistatic relationship between HELQ and XRCC2 in replication fork slowing.** Superplot of Figure 3C showing all three biological replicates in one graph. The IdU/CldU (red/green) ratio is plotted. The DNA fiber labeling scheme and representative fibers for each genetic condition are shown above each graph. At least 100 tracks were measured per condition in each of three independent experiments, which produced similar results.

**Supplementary Figure 5. HELQ acts on forks with leading-strand template ssDNA in the presence of RPA.** **(A)** SMARCAL1 was transiently downregulated in U2OS cells using siRNA and DNA fiber assay was performed as in Figure 1. **(B)** Superplot of Figure 4B showing all three biological replicates in one graph. The IdU/CldU (red/green) ratio is plotted. The DNA fiber labeling scheme and representative fibers for each genetic condition are shown above each graph. At least 100 tracks were measured per condition in each of three independent experiments, which produced similar results.

**Supplementary Figure 6. Purified HELQ promotes fork reversal with oligonucleotide-based substrates.** **(A)** Purification scheme for HELQ (left) and analysis of 1 µg purified HELQ (wild-type and K365M) by 10% SDS-PAGE with Coomassie Blue staining. **(B)** Schematic of the fork reversal substrates, including fluorescence-labeled forks with a leading- or lagging-strand gap (top and bottom, respectively). **(C)** Leading-gap (i) and lagging-gap (ii) substrates were incubated with HELQ for 30 min. Top, representative gels; bottom, quantification (error bars, SEM from three replicates). **(D)** Time dependence of HELQ-mediated fork reversal with lagging-gap substrates. Top, representative gels; bottom, quantification (error bars, SEM from three replicates). **(E)** ATP requirement for HELQ-mediated fork reversal. Wild-type or K365M HELQ was incubated with lagging-gap substrates with or without ATP as indicated. **(F)** Effect of BCDX2 on HELQ-mediated reversal of the lagging-gap fork. Reactions contained 3 nM or 6 nM HELQ and increasing concentrations of purified BCDX2, as indicated. **(G)** Effect of BCDX2 on HELQ activity on the leading-gap fork substrate. Reactions contained 3 nM or 6 nM HELQ and increasing BCDX2 concentrations as indicated. Top, representative gel; bottom, quantification showing no detectable effect of BCDX2 on product formation. Top, representative gels; bottom, quantification of reversed products (%). For (C–G), bar graphs show mean ± SEM from three independent replicates; dots indicate individual replicate values.

**Supplementary Figure 7. HELQ knockout restores replication fork stability in BRCA2- and FANCD2-deficient cells. (A)** Superplot of Figure 5B showing a combined scatter plot with medians from three biological replicates. **(B)** Representative IdU track length scatter plot corresponding to the replicate shown in Figure 5B. Median IdU track lengths (>100 fibers per condition) are indicated by horizontal lines. **(C)** Superplot of Figure 5C showing a combined scatter plot with medians from three biological replicates. Experiments were performed three times with similar results. Statistical significance was assessed by Kruskal-Wallis test: n.s., not significant; **** p-value<0.0001; *** p-value< 0.001; ** p-value< 0.01.

**Supplementary Figure 8. HELQ facilitates RAD51 fork protection against MRE11 degradation. (A)** Superplot of Figure 7A showing a combined scatter plot with medians from three biological replicates. **(B)** Model of the HELQ function at the stalled replication fork. First, HELQ is recruited to the leading strand ssDNA gap and unwinds the parental strand some distance upstream. Next, it acts on the complementary parental strand, after which the daughter strands are brought back together and are annealed.
